# AcDCXR Is a Cowpea Aphid Effector With Putative Roles in Altering Host Immunity and Physiology

**DOI:** 10.3389/fpls.2020.00605

**Published:** 2020-05-15

**Authors:** Jacob R. MacWilliams, Stephanie Dingwall, Quentin Chesnais, Akiko Sugio, Isgouhi Kaloshian

**Affiliations:** ^1^Graduate Program in Biochemistry and Molecular Biology, University of California, Riverside, Riverside, CA, United States; ^2^Department of Biochemistry, University of California, Riverside, Riverside, CA, United States; ^3^Université de Strasbourg, INRAE, SVQV UMR-A1131, Colmar, France; ^4^INRAE, UMR1349, Institute of Genetics, Environment and Plant Protection, Le Rheu, France; ^5^Department of Nematology, University of California Riverside, Riverside, CA, United States; ^6^Institute for Integrative Genome Biology, University of California, Riverside, Riverside, CA, United States

**Keywords:** cowpea aphid, salivary proteins, effector, diacetyl/L-xylulose reductase, DCXR, methylglyoxal, host defense

## Abstract

Cowpea, *Vigna unguiculata*, is a crop that is essential to semiarid areas of the world like Sub-Sahara Africa. Cowpea is highly susceptible to cowpea aphid, *Aphis craccivora*, infestation that can lead to major yield losses. Aphids feed on their host plant by inserting their hypodermal needlelike flexible stylets into the plant to reach the phloem sap. During feeding, aphids secrete saliva, containing effector proteins, into the plant to disrupt plant immune responses and alter the physiology of the plant to their own advantage. Liquid chromatography tandem mass spectrometry (LC-MS/MS) was used to identify the salivary proteome of the cowpea aphid. About 150 candidate proteins were identified including diacetyl/L-xylulose reductase (DCXR), a novel enzyme previously unidentified in aphid saliva. DCXR is a member of short-chain dehydrogenases/reductases with dual enzymatic functions in carbohydrate and dicarbonyl metabolism. To assess whether cowpea aphid DCXR (AcDCXR) has similar functions, recombinant AcDCXR was purified and assayed enzymatically. For carbohydrate metabolism, the oxidation of xylitol to xylulose was tested. The dicarbonyl reaction involved the reduction of methylglyoxal, an α-β-dicarbonyl ketoaldehyde, known as an abiotic and biotic stress response molecule causing cytotoxicity at high concentrations. To assess whether cowpea aphids induce methylglyoxal in plants, we measured methylglyoxal levels in both cowpea and pea (*Pisum sativum*) plants and found them elevated transiently after aphid infestation. Agrobacterium-mediated transient overexpression of AcDCXR in pea resulted in an increase of cowpea aphid fecundity. Taken together, our results indicate that AcDCXR is an effector with a putative ability to generate additional sources of energy to the aphid and to alter plant defense responses. In addition, this work identified methylglyoxal as a potential novel aphid defense metabolite adding to the known repertoire of plant defenses against aphid pests.

## Introduction

Cowpea (*Vigna unguiculata*) is one of the most important agronomic plant species grown in semiarid tropical regions of the world. Cowpea is well adapted to biotic and abiotic stresses and provides an excellent source of nutrition (Singh et al., 2002; Timko and Singh, 2008). However, a stress that is a limiting factor in cowpea production is infestation by the cowpea aphid, *Aphis craccivora* (Jackai and Daoust, 1986). Cowpea aphid infestation can cause devastating effects; it has been reported that young plants of highly susceptible cowpea cultivars were killed by an infestation of cowpea aphids initiated with fewer than ten aphids (Ofuya, 1995). Cowpea aphid feeding induced damage includes chlorosis, leaf curling, and stunted growth resulting in a decrease in yield (Blackman and Eastop, 2000; Kamphuis et al., 2012; Choudhary et al., 2017). In addition to cowpea aphid being a deadly pest, this aphid species is also known to vector over 50 plant viruses (Chan et al., 1991).

There are about 4500 species of aphids reported to date (Remaudiere and Remaudiere, 1997; Blackman and Eastop, 2000; Sorenson, 2009). Of these species, only 100 are considered to have an economic impact and 14 are considered to be serious pests, among which is the cowpea aphid (Sorenson, 2009). Aphids feed differently from chewing insects, which generate massive mechanical tissue damage. Aphids insert their specialized and flexible mouthparts, the stylets, through plant tissues to reach their source of food, the phloem sap, thus avoiding much of the mechanical tissue damage (Tjallingii and Esch, 1993; Tjallingii, 2006). *En route* to the phloem, aphids puncture cells and deposit saliva in the plant apoplast and the punctured cells to facilitate feeding and interfere with plant defenses (Miles, 1999; Will et al., 2007). Aphid feeding and colonization damage the plant, and aphids are categorized based on the type of damage they incur onto their hosts. Aphids that cause extensive direct damage are considered phytotoxic, whereas others that cause indirect damage – for example, by transmitting viruses – are considered non-phytotoxic (Nicholson et al., 2012). Phytotoxic aphids, such as the Russian wheat aphid (*Diuraphis noxia*) and greenbug (*Schizaphis graminum*), cause damage in low numbers and are believed to secrete salivary proteins into the plant that are responsible for the increased manifestation of the damage symptoms. In contrast, the non-phytotoxic aphids, like the pea aphid (*Acyrthosiphon pisum*) and potato aphid (*Macrosiphum euphorbiae*), do not cause damage at low numbers and secrete salivary proteins to enhance feeding and interfere with plant defenses (Nicholson et al., 2012; Nicholson and Puterka, 2014; Chaudhary et al., 2015).

Aphid saliva has been shown to contain effector proteins that are necessary for successful aphid colonization (Mutti et al., 2006, 2008; Bos et al., 2010; Atamian et al., 2013; Pitino and Hogenhout, 2013; Elzinga et al., 2014; Naessens et al., 2015; Wang et al., 2015; Will and Vilcinskas, 2015; Guy et al., 2016; Kaloshian and Walling, 2016). To characterize aphid salivary protein content, the saliva of several aphid species has been investigated with liquid chromatography tandem mass spectrometry (LC-MS/MS) (Harmel et al., 2008; Carolan et al., 2009; Cooper et al., 2010, 2011; Rao et al., 2013; Vandermoten et al., 2014; Chaudhary et al., 2015; Thorpe et al., 2016; Boulain et al., 2018; Loudit et al., 2018; Yang et al., 2018). These studies have identified numerous conserved salivary proteins common among the different aphid species as well as some that have only been identified in a single aphid species. The conserved proteins are presumed to be a core set of aphid effectors that are used by aphids to facilitate feeding or disrupt general plant defenses, while the unique proteins identified in only a single aphid species or biotype, act in a species-specific host-aphid interaction (Thorpe et al., 2016). This recent wealth of salivary protein identification stems from the release of additional aphid genomes and transcriptomes. Since the first aphid genome was released for the pea aphid, five additional aphid genomes are publicly available (International Aphid Genomics Consortium, 2010; Nicholson et al., 2015; Mathers et al., 2017; Wenger et al., 2017; Thorpe et al., 2018). Numerous aphid transcriptomes are also available including a transcriptome for the cowpea aphid (Agunbiade et al., 2013). Three main criteria have been used to identify putative aphid effectors: (1) expression of the candidate transcripts in aphid heads or salivary glands with prediction for secretion, (2) presence in saliva, and (3) sequence similarity to previously identified aphid effectors.

In general, microbial, nematode and pest effectors are diverse, lacking consensus sequences and features, making it difficult to predict effectors. This has led to reporting of mostly specific subclasses of effectors. For example, effectors from plant pathogenic fungi are small sized proteins with high cysteine content while those from Phytophthora contain a RXLR motif (Jiang et al., 2008; Stergiopoulos and de Wit, 2009; Petre and Kamoun, 2014; Sperschneider et al., 2015). To enhance plant fungal effector predictions, EffectorP was developed as a machine-learned predictor for fungal effectors that does not rely only on predetermined thresholds based on criteria including protein size and cysteine content (Sperschneider et al., 2016, 2018). It is therefore likely that the repertoire of aphid effectors can be enhanced with the development of machine learned effector identification programs.

Numerous studies have functionally characterized aphid effectors. These included overexpression of the candidate effector *in planta* or silencing it, through plant-mediated RNAi or injection with RNAi constructs, in the aphid and determining aphid performance on the plants. Of the effectors experimentally tested, about a dozen have shown altered aphid colonization phenotypes (Mutti et al., 2006, 2008; Bos et al., 2010; Atamian et al., 2013; Pitino and Hogenhout, 2013; Elzinga et al., 2014; Abdellatef et al., 2015; Naessens et al., 2015; Wang et al., 2015; Will and Vilcinskas, 2015; Guy et al., 2016; Kettles and Kaloshian, 2016). The altered survival/colonization phenotypes determined by some of these effectors act in species-specific and host-specific manner (Atamian et al., 2013; Pitino and Hogenhout, 2013; Elzinga et al., 2014; Rodriguez et al., 2017).

To date, the plant targets for only Mp1 and Me10 aphid effectors have been identified and the mechanism of effector function partially elucidated (Rodriguez et al., 2017; Chaudhary et al., 2019). The function of two additional aphid effectors MIF1 (Naessens et al., 2015) and Armet (Wang et al., 2015) have been predicted based on the function of homologous sequences from other organisms. Both MIF1 and Armet are highly conserved proteins in the animal kingdom. MIF1 encodes a macrophage migration inhibitory factor that is a cytokine deposited in aphid saliva during feeding (Calandra, 2003; Naessens et al., 2015). Armet in mammalian systems and in Drosophila has been reported in the cell as part of the unfolded protein response and extracellularly as a neurotrophic factor (Lindholm et al., 2007, 2008; Palgi et al., 2009, 2012). Both MIF1 and Armet are important for the pea aphid survival as knockdown of their expressions results in shortened lifespan (Naessens et al., 2015; Wang et al., 2015). The function of an additional effector, Me47 encoding a Glutathione S-transferase (GST), was shown based on its GST enzymatic activity and its ability to detoxify isothiocyanates that are implicated in herbivore defense (Kettles and Kaloshian, 2016).

Here we report the salivary proteome of a California population of the cowpea aphid using LC-MS/MS and publicly available aphid genomes and transcriptomes. We also characterize the function of a novel salivary protein, diacetyl/L-xylulose reductase (DCXR). DCXR is a member of short-chain dehydrogenases/reductases (Nakagawa et al., 2002). Mammalian orthologs of DCXR are involved in NADPH-dependent reduction of both carbohydrates and dicarbonyls (Nakagawa et al., 2002; Ishikura et al., 2003; Ebert et al., 2015). The reversible oxidative reduction of the carbohydrates xylitol and L-xylulose can lead to an additional energy source through the pentose phosphate pathway (Sochor et al., 1979; Nakagawa et al., 2002). The reduction of dicarbonyls detoxifies and prevents the formation of advanced glycation end-products (AGEs), also known as glycotoxins, associated with development of numerous degenerative human diseases (Chen et al., 2009; Gkogkolou and Bohm, 2012; Kizer et al., 2014). In plants, the build-up of dicarbonyls leads to oxidative stress and cell death resulting in stunted growth (Hoque et al., 2012; Ray et al., 2013; Sankaranarayanan et al., 2015; Li, 2016). One of these dicarbonyls, generated through multiple pathways in plants and animals, is methylglyoxal (Yadav et al., 2005a, b; Hoque et al., 2016; Mostofa et al., 2018). Depending on concentration, methylglyoxal can act as defense signaling molecule or as a cytotoxin during abiotic stress in plants (Li, 2016). Recently methylglyoxal has also been implicated in plant defense against biotic stresses (Melvin et al., 2017). Here we report the identification of DCXR in cowpea aphid saliva. We show that the recombinant cowpea aphid DCXR, AcDCXR, is able to catalyze the reversible xylitol to xylulose reaction as well as to utilize methylglyoxal as substrate. We also demonstrate that aphid feeding induced methylglyoxal accumulation and that expression of AcDCXR *in planta* enhanced aphid fecundity contributing to the success of the aphid as a pest.

## Materials and Methods

### Plants and Growth Condition

Cowpea California blackeye cultivar 46 (CB46) and pea (*Pisum sativum*) cv ZP1130 were grown in UC Mix 3 soil^1^ in 32 oz plastifoam cups in a pesticide free room at 22–24°C with a 16:8 light:dark photoperiod. Plants were fertilized weekly with MiracleGro (18-18-21; Stern’s MiracleGro Products).

### Aphid Colony

A colony of cowpea aphids, collected from a field in Riverside, California, in summer of 2016, was reared on cowpea cv CB46. A second colony, taken from the cowpea plants, was reared on pea cv ZP1130 for 3 months before use. The colonies was maintained separately in insect cages in growth chambers at 26–30°C with a 16:8 light:dark photoperiod. The colony on cowpea was used for aphid saliva collection and the colony on pea was used for aphid bioassays.

### Saliva Collection

Cowpea aphid saliva was collected by feeding mixed developmental stages of the aphid on a water diet as previously described (Chaudhary et al., 2015). About 100–200 mixed stage aphids were loaded in a feeding chamber, consisting of a plastic cylinder with one end containing the diet inside a parafilm sachet, and the other end secured with a cheesecloth. Aphids were allowed to feed on the 200 μL of ultrapure autoclaved water for 16 h under yellow light. The components of the chamber were sterilized or treated with alcohol and all materials were handled in a laminar flow hood using aseptic conditions. After feeding the diet was collected aseptically using a pipet and stored at −80°C. A new cohort of aphids were used for each overnight collection and saliva was collected from an estimated 10,000 aphids over a three-month period.

### Saliva Preparation for MS/MS

Saliva was vacuum concentrated down to protein pellets and dissolved in 100 μL trypsin buffer (50 mM ammonium bicarbonate, pH 8.0, 10% v/v acetonitrile) containing 1 μg trypsin and treated overnight at 37°C. After trypsin digestion, the sample was centrifuged, the supernatant was collected, pelleted with a speedvac concentrator and suspended in 24 μL 0.1% formic acid for LC-MS/MS analysis.

## LC-MS/MS

A MudPIT approach was employed to analyze the trypsin-treated samples. A two-dimension nanoAcquity UPLC (Waters) and an Orbitrap Fusion MS (ThermoFisher Scientific) were configured together to perform online 2D-nano LC-MS/MS analysis. The 2D-nanoLC was operated with a 2D-dilution method that was configured with nanoAcquity UPLC. Two mobile phases for the first dimension LC fractionation were 20 mM ammonium formate (pH 10) and acetonitrile, respectively. Online fractionation was achieved by 5-min elution off a NanoEase trap column (PN# 186003682; Waters) using stepwise-increased concentration of acetonitrile. A total of five fractions were generated with 13, 18, 21.5, 27, and 50% of acetonitrile, respectively. A final flushing step used 80% acetonitrile to clean up the trap column. Each and every fraction was then analyzed online using a second dimension LC gradient.

For the second-dimension LC, a BEH130 C18 column (1.7 μm particle, 75 μm i.d., 20 cm long, PN# 186003544; Waters) was used for peptide separation. A Symmetry C18 (5 μm particle, 180 μm i.d., 20 mm long, PN# 186003514; Waters) served as a trap/guard column for desalting and pre-concentrating the peptides for each MudPIT fraction. The solvent components for peptide separation were as follows: mobile phase A was 0.1% formic acid in water, and mobile phase B was 0.1% formic acid in acetonitrile. The separation gradient was as follows: at 0 to 1 min, 3% B; at 2 min, 8% B; at 50 min, 45% B; at 52–55 min, 85% B; at 56–70 min, 3% B. The nano-flow rate was set at 0.3 μl/min without flow-splitting.

Spectra were obtained using Orbitrap Fusion MS (Thermo Fisher Scientific). The Orbitrap Fusion MS was in positive ion mode with an ion transfer tube temperature of 275°C. The isolation window used was 2 Da. Three different types of dissociation were used: Collision Induced Dissociation (CID), High-energy Collision Induced Dissociation (HCD), and Electron Transfer Dissociation (ETD). The energy for each of these was 30%. Three scan ranges were used (300–1800, 300–2000 400–1400 Da) with 30 s dynamic exclusion.

### Proteome Data Analysis

The MS/MS spectra were filtered for high confident peptides with strict FDR (1%), with enhanced peptide and protein annotations using the software Proteome Discoverer v2.3 (Thermo Fisher). Spectra with peptide sequences less than 6 residues were removed. The search parameters allowed for 0.5 Da mass tolerance and 2 missed cleavage sites. The following modifications were included: modification of Met Oxidation ± 15.99492 D, Lys Acetyl ± 42.01057 D, Ser, Thr, Tyr Phospho ± 79.966333 D, *N*-Terminus Formyl ± 27.99492 Da, Pyro-Glu ± 17.02655 Da, *N*-Terminus Acetyl ± 42.01057 Da. The identified peptides were then searched against an aphid proteome database compiled from every aphid genome available on NCBI and AphidBase (Pea aphid, Russian wheat aphid, soybean aphid (*Aphis glycines*), bird cherry-oat aphid (*Rhopalosiphum padi*), green peach aphid (*Myzus persicae*), and black cherry aphid (*Myzus cerasi*) and other aphid proteins deposited in NCBI in 2017. These other proteins included six-frame translations of a cowpea aphid transcriptome and the transcriptome of the potato aphid). The 13,330 PSMs identified corresponded to 2,119 proteins and were further filtered to 721 protein group hits. Only high confidence (99%) were considered further filtering the protein groups to 521 protein groups. Spectra that came up when filtering out possible contaminants with a FASTA file containing common contaminants. To accept proteins, they needed to have at least 3 peptides in at least 2 of the 3 replicates (CID, HCD, ETD). The raw peptide spectra were deposited in the Mass Spectrometry Interactive Virtual Environment (MassIVE) repository with the proteome ID: PXD017323.

### Annotation

The MS/MS identified proteins were annotated with BLASTP using OmicsBox (V 1.1.135 Hotfix) and the NCBI non-redundant protein database with the taxonomy filter for aphids, Aphidomorpha (3380) (*e*-value = 1e-3) (Gotz et al., 2008). The proteins were then subjected to BLASTP to the pea aphid annotation v2.1b proteins on Aphidbase to identify the corresponding ACYPI homologs (BIPAA, 2017). Gene ontology (GO) was determined for molecular function, biological process, and cellular component using InterProScan (v5.36-75.0) (Gotz et al., 2008; Jones et al., 2014). The identified proteins were screened with SignalP (V3.0 and V5.0) and SecretomeP 1.0 using eukaryote and mammalian filters, respectively, and by TMHMM V2.0 (Krogh et al., 2001; Bendtsen et al., 2004a, b; Armenteros et al., 2019). The proteins were further analyzed using EffectorP 2.0 (Sperschneider et al., 2018).

### Clone Construction

RNA was extracted from 10 mixed developmental stage aphids using Trizol (Invitrogen), and cDNA was synthesized using SuperScript III reverse transcriptase (Invitrogen) according to manufactures instructions. Using *AcDCXR* (MN855408) gene-specific Gateway recombination primers (DCXRF- ACAAGTTTGTACAAAAAAGCAGGCTCCATGGAAGAATTC TTTGTCGGAAAAAAGTTCAT, DCXRR- GGGGACCACT TTGTACAAGAAAGCTGGGTCACTGGCCAAAAATCCACCA TC), the DCXR coding region, excluding the secretion signal peptide, was amplified using Q5^®^ High-Fidelity DNA Polymerase (New England Biolabs) with the following conditions: an initial 98°C for 30 s, 98°C for 7 s, 54°C for 20 s, 72°C for 30 s, for 30 cycles and a final cycle of 72°C for 3 min. DCXR was purified using GeneJET PCR Purification Kit (Thermo Scientific) and recombined into vector pDONR207 (Invitrogen) using BP Clonase (Invitrogen). Following Sanger sequencing pDONR207-DCXR was recombined into the expression vectors pDEST17 (Invitrogen; pDEST17-DCXR), pEAQ-HT-DEST1 (Sainsbury et al., 2009; pEAQ-HT-DEST1-AcDCXR), or pCAMBIA1300-GW-mScarlet (pCAMBIA1300-AcDCXR-mScarlet). pCAMBIA1300-GW-mScarlet was developed by modifying pCAMBIA1300 using parts from pGWB614 and p#128060 by restriction digestion and ligations. After transformation into *E. coli* strain DH5α and the purified pDEST17-DCXR was transformed into *E. coli* strain ArcticExpress (Agilent) while pEAQ-HT-DEST1-AcDCXR and pCAMBIA1300-AcDCXR-mScarlet were transformed into *Agrobacterium tumefaciens* strains AGL01 and GV3101, respectively.

### Protein Purification

The pDEST17-AcDCXR was purified in a similar manner as previously described for the aphid effector Me47 (Kettles and Kaloshian, 2016). Briefly, pDEST17-AcDCXR (*N*-terminal 6xHis tag) in ArcticExpress was grown in LB media at 37°C to an OD_600_ of 0.8 and the expression induced by adding of 0.5 mM IPTG followed by incubation at 10°C for 16 h. After centrifugation (6,000 × *g* for 20 min) the cells were resuspended in chilled lysis buffer (300 mM NaCl, 50 mM NaH_2_PO_4_/Na_2_HPO_4_, pH 7.0). The cells were lysed using sonication (4 × 15 s pulses), the soluble protein fraction was separated by centrifugation (10,000 × *g* for 45 min) and incubated with Ni- NTA agarose beads (Qiagen) for 1 h at 4°C with gentle agitation. The column was washed with the lysis buffer containing 40 mM imidazole to remove non-specifically bound proteins. After four washes, DCXR was eluted with three washes of lysis buffer containing 150, 200, and 200 mM of imidazole, respectively. The eluted fractions were concentrated with VivaSpin 500 Centrifugal Concentrator PES (Sartorius, United Kingdom) and monitored using Bradford assay with BSA as the standard. The recombinant DCXR was analyzed on a 12% SDS–PAGE using Coomassie Brilliant Blue G-250 staining.

### AcDCXR Enzyme Activity Assays

Oxidation of xylitol to xylulose by recombinant DCXR was measured through the reduction of NADP^+^ to NADPH as previously described (Yang et al., 2017) with minor modification. A 0.5 mL reaction mixture containing 10 μg AcDCXR 100 mM glycine buffer, pH 9.5, 3 mM MgCl_2_, NADP^+^, and 200 mM xylitol were used in 1 mL cuvettes and a Beckman Coulter Du^®^ 730 Life Sciences spectrophotometer. Reactions began after the addition of AcDCXR, and changes in absorbance at 340 nm were monitored. The reaction rates were calculated based on the NADP^+^ concentrations.

Methylglyoxal reduction by recombinant DCXR was measured through the oxidation of NADPH to NADP^+^ using 1 mL cuvettes as previously described (Misra et al., 1996) and the Beckman Coulter Spectrophotometer. The 0.5 mL reaction was composed of 10 μg DCXR, 100 μM sodium phosphate buffer (NaH_2_PO_4_/Na_2_HPO_4_, pH 6.5), 200 μM NADPH and methylglyoxal. The reaction was initiated with the addition of NADPH and monitored by the decrease in absorbance at 340 nm. The reaction rates were calculated based on the methylglyoxal concentrations.

### Transient Expression in Pea and Western Blot Analysis

*Agrobacterium tumefaciens* strain AGL01, carrying either pEAQ-HT-GFP or pEAQ-HT-DEST1-AcDCXR, were used in transient expression of pea, *Pisum sativum*, cv. ZP1130 as described previously (Guy et al., 2016). Bacterial cells, grown up overnight in YEP media, were harvested, washed three times in infiltration buffer (10 mM MgCl_2_, 10 mM MES pH 5.6, and 150 μM acetosyringone) and resuspended at a final OD_600_ of 0.5. The youngest expanded leaf of a 2-week-old plant was infiltrated with a needleless syringe.

The duration of GFP expression in pEAQ-HT-GFP infiltrated leaves was monitored with Western blot analysis. Three 1 cm diameter leaf disks were cut from the same agroinfiltrated leaf using a cork borer after 2, 3, 5, 7, 8, 9, and 10 days post infiltration. Protein was extracted from the leaf disks by grinding in 200 μl lysis buffer (6 M Urea, 2 M Thiourea, 1% Protease inhibitor cocktail [Sigma P9599]). Samples were centrifuged at 14,000 × *g* for 5 min and the supernatant was resuspended in equal volume 2x loading buffer (100 mM Tris-HCl pH 6.8, 100 mM DTT, 10% glycerol, 2% SDS, 0.01% bromophenol blue). About 25 μg of protein were loaded per sample on 12% SDS–PAGE and transferred to a nitrocellulose membrane. The membrane was probed with mouse anti-GFP antibody (Sigma) and secondary antibody, goat anti-mouse HRP-conjugated (Santa Cruz Biotechnology). Primary antibody was used at 1:2000 and secondary antibody was used at 1:2000 dilution. Pierce ECL Western Blotting Substrate (Thermo Scientific) was used to detect the signal with autoradiography film (Denville Scientific Inc.).

### *in planta* Localization of AcDCXR

*Agrobacterium tumefaciens* GV3101 carrying pCAMBIA1300-DCXR-mScarlet or pCAMBIA1300-GFP were grown and prepared as previously described for transient agroexpression. At an OD_600_ = 0.5 each, the constructs were co-infiltrated in *Nicotiana benthamiana* leaves. Three days post infiltration, leaf epidermal cells were analyzed using a Leica SP5 confocal microscope. GFP and mScarlet were excited by 488 nm and 543 nm filters, respectively, and images were collected through band emission filters at 498–520 nm and 553–650 nm, respectively.

### Aphid Bioassays

A day after agroinfiltration, five adult cowpea aphids were caged onto the adaxial side of an agroinfiltrated leaf of 2-week-old pea plants. After 24 h (i.e., 2 days post infiltration; dpi), the adult aphids were removed, and 5 to 6 new-born nymphs were left on the leaf with both the adaxial and abaxial sides of the leaf accessible to the aphids. Eight days later (10 dpi), the surviving aphids were counted and transferred to a new infiltration site on a plant infiltrated 2 days earlier. The fecundity of these aphids was monitored two and five days later (i.e., when the aphids were 12 and 15 day-old). The nymphs were removed after each counting. This experiment was performed three times. Each experiment consisted of 13–15 plants per construct. All experiments were conducted at 22°C, 16:8 light:dark photoperiod.

### Determining Methylglyoxal Levels

Methylglyoxal levels were evaluated in 2-week-old cowpea and pea plants following the protocol by Borysiuk et al. (2018). Highly infested leaves were harvested at day 1, 2, and 3 after infestation. Briefly, samples were homogenized in 5% perchloric acid and centrifuged at 13,000 × *g* for 10 min at 4°C. The supernatant was decolorized with charcoal and neutralized with 1 M potassium carbonate. After centrifuging at 13,000 × *g* at 4°C the supernatant was used to estimate the methylglyoxal concentration in sodium dihydrogen phosphate buffer (pH 7.0). The absorbance was recorded after 10 min incubation with *N*-acetyl-L-cysteine to monitor the N-a-acetyl-S-(1-hydroxy-2-oxo-prop-1-yl) cysteine formation (Wild et al., 2012). Methylglyoxal concentration was determined using a standard curve of known methylglyoxal concentrations. The experiment with pea was performed once and with cowpea was performed twice.

### Statistical Analyses

We used generalized linear models (GLM) with a likelihood ratio and chi-square test to assess whether AcDCXR expression had an effect on aphid survival and fecundity. Data on aphid survival were analyzed with GLM following a binomial distribution and data on aphid fecundity were assumed to follow a Poisson distribution. The fit of all generalized linear models was checked by inspecting residuals and QQ plots. Methylglyoxal levels in plants were analyzed using a nested ANOVA (biological replicates treated as random factor) (package R: ‘nlme’). When a significant effect was detected, a pairwise comparison using multiple comparisons of the means (package R: ‘multcomp’) (Tukey contrasts, *p*-values adjustment with ‘fdr’ method) at the 0.05 significance level was used to test for differences between days after infestation. Statistical analyses were performed using the R software (version 3.6.0) (R Core Team, 2019).

## Results

### Aphid Salivary Proteome Analyses and Annotation

To identify the protein composition of the cowpea aphid saliva, aphid saliva was collected in parafilm feeding pouches containing water. The contents of the pouches were concentrated and subjected to proteome analyses. The peptides identified by LC-MS/MS were searched against a custom aphid protein database. The database was composed of proteomes based on all aphid genomes available in the summer of 2017, as well as cowpea aphid-specific transcriptome and a transcriptome from the potato aphid, both with six-frame translations. Around 175 candidate proteins were identified with at least three peptides from at least two replicates and having at least one unique peptide (Supplementary Table 1). The identified proteins were then annotated using BLASTP with OmicsBox (TaxID: Aphididiae 27482). Among these annotated proteins, 18/175 (10.29%) were uncharacterized. In addition, functional redundancies were recorded among the proteins with annotations. To eliminate these redundancies, the proteins were subjected to BLASTP on AphidBase to identify their corresponding ACYPI homolog using the pea aphid protein database annotation v2.1b. Among these proteins, 47/175 (26.86%) shared one of 21 ACYPI top hits. Although these 47 proteins had at least one unique peptide, we grouped them as 21 proteins, resulting in a total of 149 salivary proteins (Supplementary Table 1).

Annotation of these proteins presented a wide range of functional attributes to the cowpea aphid salivary proteins. Among the 149 identified proteins, 33 proteins with similar functional annotations have been previously reported in the saliva of a cowpea aphid population from Gabon, Africa (Loudit et al., 2018). Among these 33 proteins are glucose dehydrogenases, carbonic anhydrases and a trehalase (Supplementary Table 1).

**FIGURE 1:**
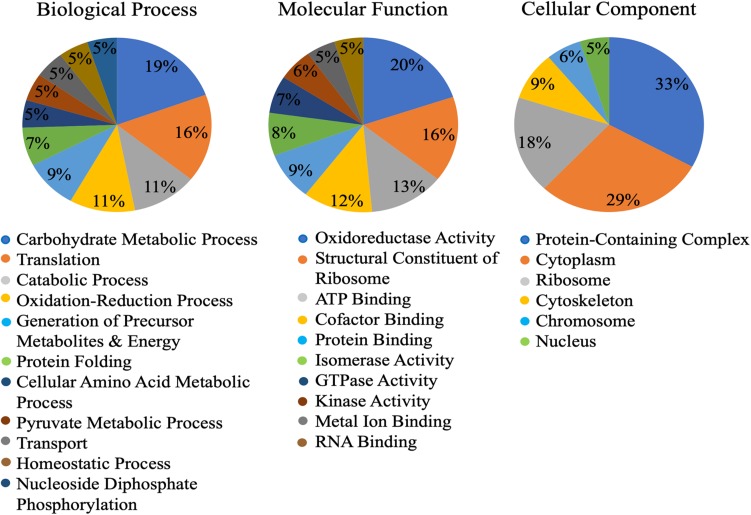
Gene ontology (GO) of the Cowpea aphid salivary proteins. Cowpea aphid salivary proteins were identified by LC-MS/MS and protein content were determined using a number of aphid genomes and the transcriptomes of cowpea aphid and potato aphid.

Of the 149 identified cowpea aphid proteins, gene ontology (GO) assigned 123 proteins with at least one GO term in the three most common ontological designations: molecular function, biological process and cellular component. The three most abundant biological process designations were carbohydrate metabolic process (19%), translation (16%) and catabolic process (11%) (Figure 1). The three most abundant molecular function designations were oxidoreductase activity (20%), structural constituent of ribosome (16%) and ATP binding (13%) (Figure 1). As for the most abundant cellular component designations, they were for protein-containing complexes (33%) and cytosol (29%) (Figure 1).

### Effector Prediction

Since the cowpea aphid genome has not been sequenced, homologous proteins from the different aphid species or those based on cowpea aphid transcriptome, used in our custom database, were used for these analyses. Multiple bioinformatics tools were harnessed to screen the identified salivary proteins for putative effector function. First, the salivary proteins were evaluated for secretion using tools that predict classical and non-classical secretions, SignalP and SecretomeP (Bendtsen et al., 2004a, b; Armenteros et al., 2019), respectively. Using SignalP, a secretion signal was detected in 29 (19.46%) proteins, while SecretomeP predicted the secretion of an additional 23 (15.44%) of the 149 salivary proteins (Table 1). To eliminate proteins with transmembrane domains, presence of transmembrane helices was evaluated using TMHMM (Sonnhammer et al., 1998). Six of these predicted secreted proteins contained transmembrane helices.

**TABLE 1:** Cowpea aphid salivary proteins identified for secretion or effector function using bioinformatic programs.

Accession	ACYPI	Description (BLASTP)	SignalP	SecretomeP	EffectorP
GAJW01000730.1_3	009769	glyceraldehyde-3-phosphate dehydrogenase [*Aphis gossypii*]			X
AG009127-PA	088094	histone H4-like, partial [*Sipha flava*]		X	X
NP_240299.1	085083	protein lin-28 homolog isoform X1 [*Sipha flava*]			X
AG010231-PA	23752	carbonic anhydrase 2-like [*Aphis gossypii*]	X		X
GAJW01000939.1_3	006727	triosephosphate isomerase [*Aphis gossypii*]			X
GAJW01000401.1_4	000057	putative diacetyl/L-xylulose reductase [*Aphis citricidus*]	X		X
Rpa07060.t1-protein	000028	nucleoside diphosphate kinase-like isoform X1 [*Rhopalosiphum maidis*]			X
Rpa13763.t1-protein	004622	probable pseudouridine-5’-phosphatase [*Rhopalosiphum maidis*]			X
GAJW01000399.1_1	001643	ribosomal protein S19e-like [*Acyrthosiphon pisum*]		X	X
NP_239859.1	000693	co-chaperonin GroES [*Buchnera aphidicola* str. APS (*Acyrthosiphon pisum*)]		X	X
GAJW01002612.1_4	002607	ras-related protein Rab-7a-like [*Rhopalosiphum maidis*]			X
Rpa11900.t1-protein	003541	peptidyl-prolyl cis-trans isomerase-like [*Aphis gossypii*]			X
GAJW01001525.1_4	008224	Uncharacterized protein LOC114123729 [*Aphis gossypii*] (Me10/Mp58)	X		X
GAJW01005083.1_5	006909	dihyropteridine reductase *[Aphis gossypii*]			X
GAJW01000315.1_5	000041	cytochrome c-like [*Aphis gossypii*]			X
GAJW01000773.1_5	000058	twinstar [*Acyrthosiphon pisum*]		X	X
GAJW01001573.1_3	007471	Superoxide dismutase [Cu-Zn] [*Aphis gossypii*]			X
AG013923-PA	006002	protein dj-1beta-like isoform X2 [*Melanaphis sacchari*]			X
AG015946-PA	26018	uncharacterized protein LOC114132136 [*Aphis gossypii*]		X	X
GAJW01000973.1_1	000031	ribosomal protein S12 [*Acyrthosiphon pisum*]			X
AG001995-PA	000422	apolipophorins-like [*Aphis gossypii*]	X		
AG007466-PA	002298	soluble trehalase [*Aphis glycines*]	X		
AG008787-PA	000288	glucose dehydrogenase [FAD, quinone]-like [*Aphis gossypii*]	X		
AG009504-PA	005582	glucose dehydrogenase [FAD, quinone]-like [*Melanaphis sacchari*]	X		
Rpa18947.t1-protein	009964	endochitinase [*Rhopalosiphum maidis*]	X		
AG005369-PA	000986	uncharacterized protein LOC114133079 [*Aphis gossypii*]	X		
AG011655-PA	009915	endoplasmin homolog [*Aphis gossypii*]	X		
AG005025-PA	002622	calreticulin [*Aphis gossypii*]	X		
GAJW01001996.1_3	009755	protein disulfide-isomerase [*Melanaphis sacchari*]	X		
Rpa07735.t1-protein	37407	uncharacterized protein LOC113549759 [*Rhopalosiphum maidis*]	X		
Rpa04366.t1-protein	005594	protein disulfide-isomerase A3-like [*Rhopalosiphum maidis*]	X		
AG011982-PA	004904	uncharacterized protein LOC114121223 [*Aphis gossypii*]	X		
AG008256-PA	004432	uncharacterized protein LOC114132390 [*Aphis gossypii*]	X		
Rpa01411.t1-protein	008926	protein disulfide-isomerase A6 homolog [*Rhopalosiphum maidis*]	X		
AG011980-PA	25151	uncharacterized protein LOC107882155 [*Acyrthosiphon pisum*]	X		
AG001228-PA	001479	phospholipase A1-like [*Aphis gossypii*]	X		
AG014046-PA	000975	calumenin-A-like [*Aphis gossypii*]	X		
AG001716-PA	007690	uncharacterized protein LOC100166851 precursor [*Acyrthosiphon pisum*]	X		
AG000496-PA	50398	RCC1 domain-containing protein 1 [*Aphis gossypii*]	X		
AG002007-PA	003669	puromycin-sensitive aminopeptidase isoform X2 [Aphis gossypii]	X		
AG012271-PA	000817	peroxidase-like [Aphis gossypii]	X		
GAJW01000521.1_1	008165	60S ribosomal protein L4 [Rhopalosiphum maidis]	X		
GAJW01002804.1_6	000056	calmodulin [*Bombyx mori*]		X	
MYZPE13164_0_v1.0_000072220.1	086010	histone H2B [*Myzus persicae*]		X	
AG009875-PA	007671	histone H3-like [*Diuraphis noxia*]		X	
AG015809-PA	003625	citrate synthase 1 [*Aphis gossypii*]		X	
Rpa01379.t1-protein	007342	annexin B9-like isoform X2 [*Rhopalosiphum maidis*]		X	
GAJW01000299.1_6	002483	eukaryotic initiation factor 4A [*Acyrthosiphon pisum*]		X	
GAOM01000916.1_4	000087	bicaudal [*Acyrthosiphon pisum*]		X	
BAH71147.1	010042	40S ribosomal protein S7-like *[Acyrthosiphon pisum*]		X	
AG001482-PA	002577	adenylate kinase isoenzyme 1 [*Aphis gossypii*]		X	
Mca25739.t1-protein	006705	ump-cmp kinase isoform X2 [*Myzus persicae*]		X	
AG001456-PA	009018	inositol monophosphatase 1-like [*Aphis gossypii*]		X	
AG004283-PA	21663	lipase member H-B-like isoform X1 [*Aphis gossypii*]		X	
GAJW01001081.1_4	004620	complement component 1 Q subcomponent-binding protein, mitochondrial [*Aphis gossypii*]		X	
AG012940-PA	006879	importin-5 [*Aphis gossypii*]		X	
GAJW01001854.1_3	009959	protein lethal(2)essential for life [*Aphis gossypii*]		X	
Mca14412.t1-protein	000100	40s ribosomal protein s4 [*Diuraphis noxia*]		X	

**FIGURE 2:**
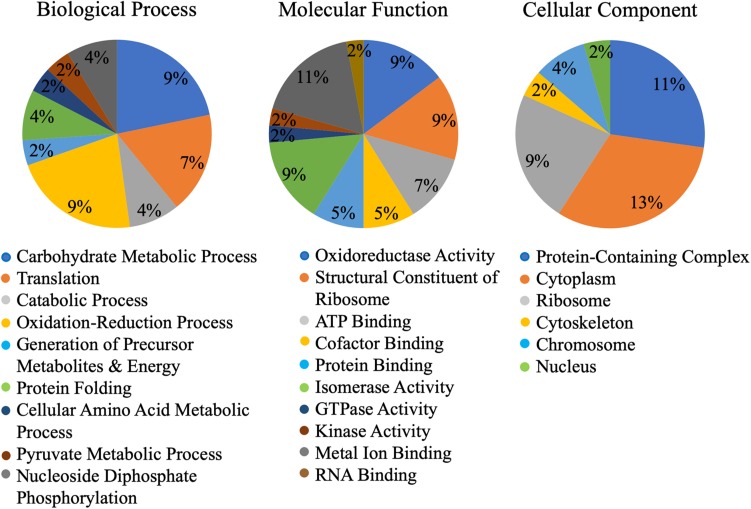
Gene ontology (GO) of putative Cowpea aphid effectors. Cowpea aphid putative effectors were identified by analyzing the salivary proteins with SignalP, SecretomeP, and EffectorP.

A machine learning approach was recently used to develop novel prediction program for fungal effectors (Sperschneider et al., 2016, 2018). We wondered whether this tool, EffectorP, could be used to predict aphid effectors. To test this, we first subjected known aphid effectors for EffectorP analysis. We tested the C002 effector, identified first in pea aphid (Mutti et al., 2008), and Me10, identified in potato aphid (Atamian et al., 2013). Both C002 and Me10 were identified as effectors by EffectorP indicating that EffectorP can be utilized as a tool to screen for aphid effectors. Using EffectorP, 20/149 (13.4%) of the cowpea aphid salivary proteins were identified as putative effectors (Table 1). Only eight of the 20 were identified for secretion by SignalP or SecretomeP. Taken together 58 proteins were predicted for secretion or for effector function encoding a wide range of functions with eight being unknowns (Figure 2 and Table 1)

### Selection and *in vitro* Characterization of AcDCXR

A set of criteria were applied to choose a putative effector protein identified by EffectorP for functional characterization. These included a previously unidentified effector predicted for secretion or with secretion signal peptide, a protein with predicted enzymatic activity, and high abundance in cowpea aphid saliva based on the SEQUEST score. Based on these criteria, DCXR was selected for further analysis.

Sequence prediction indicated that cowpea aphid DCXR (AcDCXR; GAJW01000401.1) consists of at least 263 amino acids, with the first 23 amino acids encoding a predicted signal peptide, and a conserved enzymatic domain for short-chain dehydrogenases/reductases (Supplementary Figure 1). Using AcDCXR in BLASTP searches identified DCXR homologs in seven aphid species. Interestingly, only the DCXR from cotton melon aphid (*Aphis gossypii;* XP_027848224.1) contains a secretion signal peptide (Supplementary Figure 1). Consistent with this information, DCXR has been reported previously from other aphid species but has not been previously identified in aphid saliva (Nguyen et al., 2008, 2009; Pinheiro et al., 2014).

Diacetyl/L-xylulose reductase is a multifunctional enzyme. Mammalian orthologs of DCXR have been shown to function in the glucuronic acid/uronate cycle, in a reversible reaction either oxidizing or reducing xylitol and xylulose, respectively (Sochor et al., 1979; Yang et al., 2017), as well as having α-β-dicarbonyl reductase activity to metabolize toxic carbonyls like methylglyoxal (Ebert et al., 2015). Direct comparison between AcDCXR and XP_027848224.1 showed 100% identity at the amino acid level with perfect conservation of the enzyme active site (Supplementary Figure 1). To test whether AcDCXR has similar functions as the mammalian orthologs, we expressed recombinant AcDCXR and performed enzymatic assays.

**FIGURE 3:**
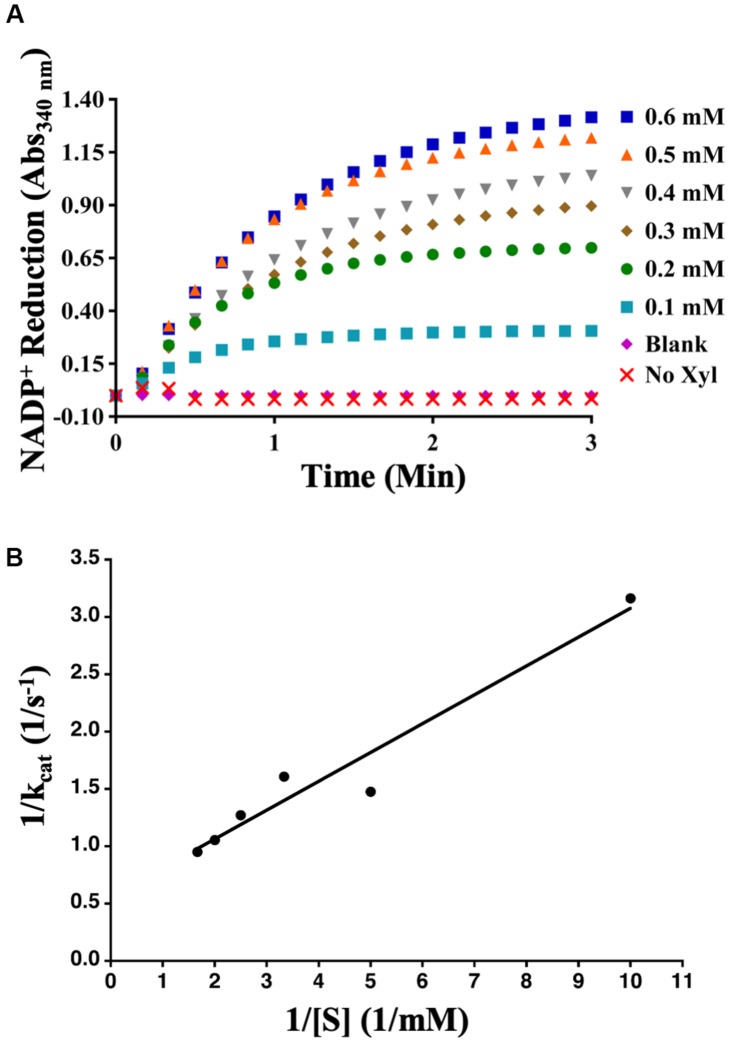
Recombinant AcDCXR oxidation activity. Xylitol oxidation by cowpea aphid recombinant AcDCXR. **(A)** Various concentrations of NADP^+^ were used to oxidize 200 mM xylitol in the presence of 10 μg of AcDCXR. Reactions containing no AcDCXR (Blank) or no xylitol (Xyl) were used as controls. **(B)** Lineweaver-Burk plot of xylitol oxidation. Data represent average of two technical replicates from a single experiment. The experiment was repeated once with similar results.

Aphid diacetyl/L-xylulose reductase, amplified from cDNAs developed from the whole bodies of mixed stages of the aphid, was cloned into the pDEST17 expression vector and expressed in *E. coli* strain ArcticExpress. Purified AcDCXR (Supplementary Figure 2) was used in two distinct enzymatic assays to check its functionality. To verify whether AcDCXR is able to oxidize xylitol to xylulose, AcDCXR was assayed using xylitol as the substrate and NADP^+^ as co-substrate. The reduction of NADP^+^ to NADPH was spectroscopically monitored by the increase of absorbance at 340 nm. AcDCXR was able to oxidize xylitol to xylulose in a NADP^+^ concentration-dependent manner (Figure 3A). Analysis of the Lineweaver-Burke plot data determined the enzymatic constants to be: *k*_*cat*_ = 1.85 s^–1^, a Km = 0.56 mM and a *V*max = 79.4 μM/min (Figure 3B).

**FIGURE 4:**
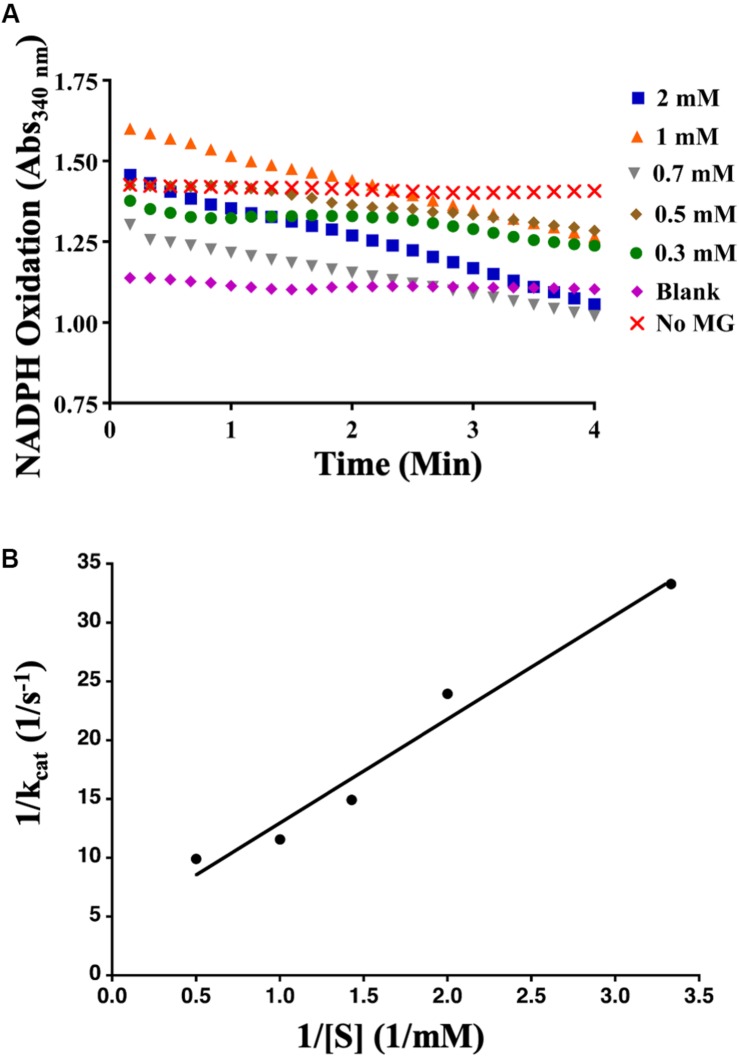
Recombinant AcDCXR reduction activity. Methylglyoxal reduction by cowpea aphid recombinant AcDCXR. **(A)** Various concentrations of methylglyoxal were reduced with 200 mM NADPH in the presence of 10 μg of AcDCXR. Reactions containing no AcDCXR (Blank) or no methylglyoxal (MG) were used as controls. **(B)** Lineweaver-Burk plot of methylglyoxal reduction. Data represent average of two technical replicates from a single experiment. The experiment was repeated once with similar results.

To determine whether AcDCXR was able to use methylglyoxal as a substrate, we tested the reduction of methylglyoxal by spectroscopically measuring the decrease in absorption of concomitant NADPH oxidation at 340 nm. We found that AcDCXR was able to reduce methylglyoxal in a concentration-dependent manner (Figure 4A). Analysis of the Lineweaver-Burke plot data determined the enzymatic constants to be: *k*_*cat*_ = 0.23 s^–1^, a Km = 1.3 mM and a *V*max = 13.8 μM/min (Figure 4B). The control reactions, in the presence of AcDCXR and absence of a substrate, showed neither oxidation nor reduction (Figures 3A, 4A). Similarly, the control reactions in the absence of the enzyme showed neither oxidation nor reduction, indicating the AcDCXR’s presence was necessary to complete the reactions (Figures 3A, 4A). The kinetic constants in AcDCXR show that, *in vitro*, it was more efficient oxidizing xylitol with a *k*_*cat*_/K_*m*_ of 3.32 mM^–1^ s^–1^ compared to reducing methylglyoxal that had only a *k*_*cat*_/K_*m*_ of 0.174 mM^–1^ s^–1^, nearly a 20-fold difference in activity.

### Functional Analysis of AcDCXR *in planta*

To functionally evaluate the role of AcDCXR on cowpea aphid colonization, AcDCXR was cloned into the binary vector pEAQ-DEST1 for Agrobacterium-mediated transient expression. Since Agrobacterium-mediated transient expression in cowpea has not yet been developed, pea plants were used for this experiment. Pea is a host for cowpea aphid and has been previously used successfully in transient expression experiments for evaluation of aphid effectors (Guy et al., 2016). Using the same cultivar of pea cv ZP1130, we first transiently expression GFP using *A. tumefaciens* strain AGL01. Monitoring GFP expression by western blot analysis, GFP was detected as early as 2 days after agroinfiltration and lasted at least for 10 days (Supplementary Figure 3). Based on the GFP expression in pea, a cowpea aphid bioassay was developed.

**FIGURE 5:**
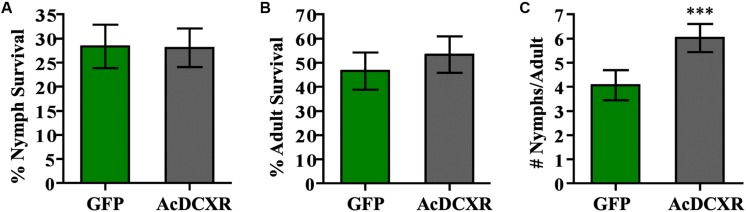
AcDCXR effect on aphid performance. *Agrobacterium tumefaciens* strain AGL01 was used to transiently express pEAQ-HT-DEST1-GFP and pEAQ-HT-DEST1-AcDCXR in *Pisum sativum* cv. ZP1130. Adult cowpea aphid adults were placed the infiltration site to lay nymphs and removed 24 h later. **(A)** The survival rate of the nymphs after 8 days on the site of infiltration. **(B,C)** A single adult was transferred to a new infiltration site of the same construct and the **(B)** survival of the adult and **(C)** fecundity were monitored. Graphs show the mean with error bars representing ± SE of the mean for *n* = 43 for GFP and *n* = 45 for AcDCXR from three independent experiments. ^∗∗∗^*P* < 0.001 as determined by generalized linear models (GLM).

**FIGURE 6:**
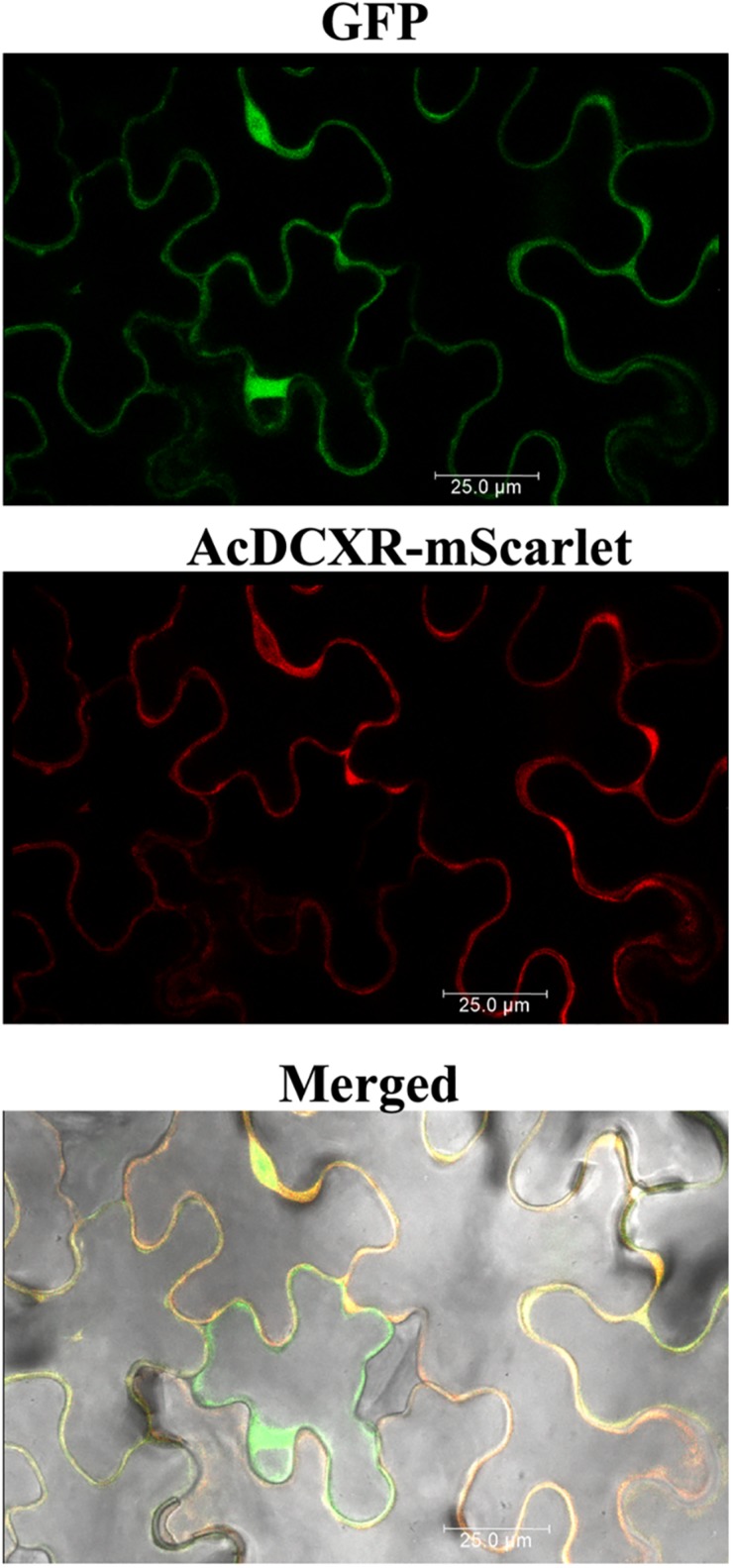
*In planta* subcellular localization of the recombinant AcDCXR. *A. tumefaciens* strain GV3101 containing pCAMBIA-1300-GFP or pCAMBIA-1300-AcDCXR-mScarlet were co-infiltrated into *N. benthamiana* leaves. Three days after agroinfiltration, leaf epidermal cells were used in confocal microscopy.

Aphid bioassays were performed to evaluate the effect of AcDCXR overexpression in pea plants on cowpea aphid. Plants were agroinfiltrated with AcDCXR or GFP control constructs as described earlier for the western blot analysis. A day post infiltration (dpi), adult cowpea aphids, maintained on pea cv ZP1130, were placed on a leaf, at the site of the infiltration, in a clip cage. After 24 h (2 dpi), all adult aphids were removed and six newborn nymphs were left at the infiltration site. At ten dpi, similar number of aphids were counted on GFP and AcDCXR infiltrated leaves indicating no effect on nymph survival rate (GLM, Chisq = 0.034, *P* = 0.854) (Figure 5A). To evaluate the fecundity of these aphids, one aphid per cage was transferred to a freshly agroinfiltrated (2 dpi) plant, with the same construct, and aphid survival and fecundity was monitored 4 and 7 days later. Sixteen days after initiation of the aphid bioassay, no difference in adult survival was detected between aphids feeding on AcDCXR compared to those feeding on the GFP infiltrated leaves (GLM, Chisq = 0.367, *P* = 0.544) (Figure 5B). However, a significant difference (GLM, Chisq = 16.901, *P* < 0.001) in aphid fecundity was observed between the aphids feeding on AcDCXR compared to those feeding on the GFP control indicating a role for AcDCXR in cowpea aphid colonization (Figure 5C). To determine the subcellular localization of AcDCXR *in planta*, AcDCXR was cloned into the binary vector pCAMBIA-1300-mScarlet and used in Agrobacterium-mediated transient expression in *N. benthiamana*. pCAMBIA-1300-AcDCXR-mScarlet was co-infiltrated with a GFP construct. As expected, GFP was detected throughout the cell including the nucleus, while AcDCXR-mScarlet was localized to the cytoplasm (Figure 6).

### Aphid Induce Methylglyoxal Accumulation

**FIGURE 7:**
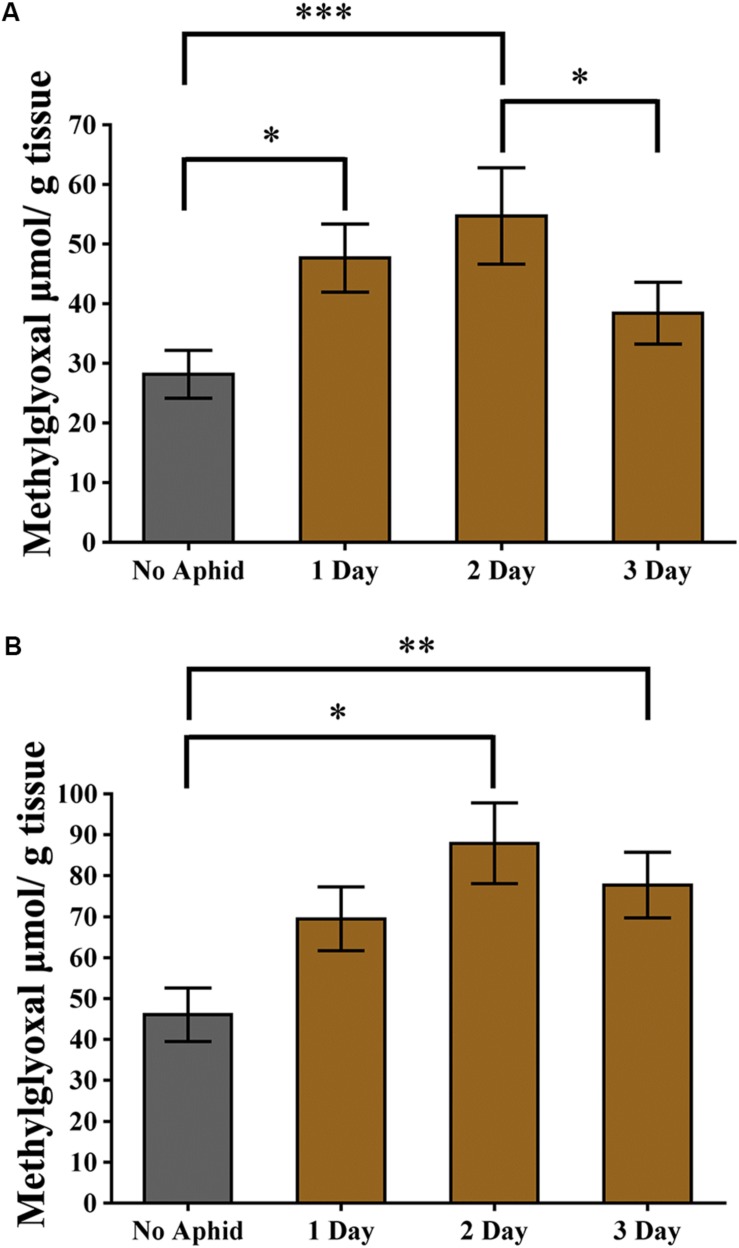
Methylglyoxal levels induced by aphid infestation. **(A)** Cowpea and **(B)** pea plants were exposed to a heavy infestation of cowpea aphids. Leaves were harvested at 1, 2, and 3 days post infestation. Uninfested plants of the same age were used as controls. Graphs show the mean with error bars representing ± SE of the mean of *n* = 6 for cowpea, from two independent experiments, and *n* = 3 for pea, from a single experiment, with two technical replicates each. ^∗^*P* < 0.05, ^∗∗^*P* < 0.01, and ^∗∗∗^*P* < 0.001 as determined by nested ANOVA followed by multiple comparisons of means.

Methylglyoxal has been shown to accumulate in multiple plant species when exposed to abiotic stresses (Yadav et al., 2005a; Hossain et al., 2009; Mustafiz et al., 2014). Recently, it was also shown that methylglyoxal accumulates in plants exposed to biotic stresses (Melvin et al., 2017). To assess if methylglyoxal also accumulates by aphid infestation, methylglyoxal levels in cowpea and pea plants were monitored. A day after infestation of cowpea plants to cowpea aphids, a significantly higher (multiple comparisons, *z* = 2.812, *P* = 0.015) levels of methylglyoxal were detected in the infested leaves compared to the uninfested control leaves (Figure 7A). Methylglyoxal levels remained significantly higher (multiple comparisons, *z* = 3.832, *P* < 0.001) on day 2 but reduced to pre-infective levels on day 3 (multiple comparisons, *z* = 1.479, *P* = 0.208) (Figure 7A). A similar trend of methylglyoxal accumulation was detected in pea leaves exposed to cowpea aphids indicating that cowpea aphid feeding induces methylglyoxal levels irrespective of the host species (Figure 7B).

## Discussion

### Cowpea Aphid Salivary Proteome

We carried out proteomics analysis to identify the salivary protein composition of a population of cowpea aphid from California. The identified proteins had a diverse range of functions including some that are uncharacterized. We were conservative in assessing the salivary proteome and used strict cut-off measures to identify the proteins. Nevertheless, we identified 149 non-redundant proteins. Previously, the salivary proteome from an African cowpea aphid population was reported (Loudit et al., 2018). The majority of the proteins identified in our study were not reported from this African population suggesting that our approach allowed us to identify higher numbers of proteins. While the cowpea aphid saliva in this work was collected in water, the African cowpea aphid saliva was collected in a sucrose-based diet and required clean up steps before undergoing mass spectrometry and that could have contributed to the low number of proteins identified in the saliva. Interestingly, both studies did not identify a set of functionally characterized aphid effectors such as Armet, Me23, Ap25, Mp2, Mp55 (Atamian et al., 2013; Pitino and Hogenhout, 2013; Elzinga et al., 2014; Wang et al., 2015; Guy et al., 2016). While in our study we identified Me10/Mp58 and SHP, the structural sheath protein, these two proteins were not identified in the African cowpea aphid saliva (Carolan et al., 2009; Chaudhary et al., 2015). The well characterized effector C002, was reported in the African population and not in this work (Mutti et al., 2006, 2008; Pitino and Hogenhout, 2013; Elzinga et al., 2014; Loudit et al., 2018). Although peptides for C002 and two additional effectors, Mp1 and MIF1, were detected in the saliva of the California cowpea aphids, this work, they did not fulfil the criteria used in our selection (Harmel et al., 2008; Naessens et al., 2015).

Unlike the salivary proteome of the African cowpea aphid, there were no proteins identified from secondary symbionts in the California cowpea aphid saliva (Loudit et al., 2018). The only bacterial proteins identified in the California cowpea aphid salivary proteome were from the primary endosymbiont *Buchnera aphidicola*, the chaperonin GroEL and GroES. GroEL has been previously identified in the saliva of several aphid species including the cowpea aphid (Chaudhary et al., 2014, 2015; Vandermoten et al., 2014; Loudit et al., 2018). GroEL is an aphid-associated molecular pattern triggering immune responses in plants (Chaudhary et al., 2014).

Our work was limited by the absence of a cowpea aphid genome and a gland/head specific transcriptome that could have been used for the peptide searches. In addition, homologous sequences from different aphid species were used in the secretion prediction analyses including some originating from transcriptomes that could have been truncated. Therefore, the number of proteins predicted for secretion, 46 out of 149 (30.9%), based on the bioinformatic programs SignalP and SecretomeP, are likely an underestimate (Table 1). Previous work describing salivary proteome from aphids with genome sequences and gland/head specific RNAseq generated sequences, also identified a large number of proteins from aphid saliva, collected in sugar and amino acid-based diets, with no prediction for secretion (Thorpe et al., 2016; Boulain et al., 2018). Boulain et al. (2018) reported 37/51 (72.5%) of the pea aphid salivary proteins with a secretion prediction. Thorpe et al. (2016), studying three different aphid species, green peach aphid, black cherry aphid, and bird cherry-oat aphid, reported only 61/204 (30%) secretion prediction of the identified salivary proteins. Taken together, this information indicates that the current bioinformatic prediction programs are likely limited in their ability to identify aphid secreted proteins.

### Effector Prediction

Here we reported the use of a machine learning plant-pathogenic fungi effector prediction program, EffectorP, for prediction of aphid effectors (Sperschneider et al., 2016, 2018). We confirmed the use of EffectorP as a possible program for identifying aphid effector proteins by successfully subjecting the well-characterized aphid effectors C002 and Me10 to EffectorP analysis (Mutti et al., 2008; Atamian et al., 2013; Pitino and Hogenhout, 2013; Chaudhary et al., 2019). Interestingly, EffectorP predicted 20/149 of the cowpea aphid proteins as effectors. Among these 20 proteins, is the functionally characterized Me10 effector and three proteins which have been predicted for effector function (Atamian et al., 2013; Elzinga et al., 2014; Thorpe et al., 2016; Chaudhary et al., 2019). Orthologs of Me10 have been identified in multiple aphid species. Me10 has been detected in plant tissues fed on by aphids and expression of Me10 in plants has been shown to enhance the performance of potato aphid on tomato and green peach aphid on *N. benthamiana* (Atamian et al., 2013; Chaudhary et al., 2015, 2019). In addition, Me10 was shown to interact with the tomato scaffold protein Fourteen-Three-Three isoform 7 (TFT7) and predicted to interfere with a mitogen-activated protein kinase defense signaling pathway (Chaudhary et al., 2019).

The remaining three previously predicted putative effectors are carbonic anhydrase, superoxide dismutase, and peptidyl-prolyl cis-trans isomerase (PPIase). The latter two proteins were identified in the proteomes of the pea aphid salivary glands (Carolan et al., 2011). While carbonic anhydrases have been identified in aphid saliva, superoxide dismutase and PPIase have not been previously reported in aphid saliva (Rao et al., 2013; Nicholson and Puterka, 2014; Chaudhary et al., 2015; Loudit et al., 2018). A carbonic anhydrase and a superoxide dismutase have been shown to be under positive selection further implicating these proteins as effectors (Thorpe et al., 2016). While clear roles for carbonic anhydrases and PPIases have not been characterized in plant immune responses, superoxide dismutases are attributed to detoxify reactive oxygen species (ROS), the well-known defense signaling molecule.

Among the EffectorP identified putative effector proteins, that had not been previously identified in aphid saliva or as a putative effector, is AcDCXR (Table 1, Supplementary Table 1). DCXR has been identified in the pea aphid salivary gland but has not been reported in the saliva of this aphid species (Carolan et al., 2011; Boulain et al., 2018). Interestingly, pea aphid homolog of AcDCXR as well as homologs from five additional aphid species with genome sequences, do not have a secretion signal peptide. The homolog from the cotton melon aphid does have a secretion signal suggesting that DCXR is one of the differential pest arsenals utilized by a subset of aphid species. An increase in DCXR accumulation was reported in a virulent biotype of greenbug infesting resistant wheat (Pinheiro et al., 2014). Additionally, enhanced accumulation of DCXR in response to heat/UV stress as well as predation by parasitoids in the potato aphid were reported from whole insects (Nguyen et al., 2008, 2009). Taken together, these information suggest that aphids may have evolved different roles for DCXR to deal with stress conditions in the plant and within the aphid itself.

### Diacetyl/L-Xylulose Reductases

In mammals DCXRs are reported to be oxidoreductases for monosaccharides and dicarbonyls. Human DCXR was first discovered while investigating the disease pentosuria and found that an enzymatic defect in DCXR was the cause of the high excretion of L-xylulose. This lead to the conclusion that L-xylulose is a possible substrate of DCXR (Wang and Van Eys, 1970). DCXR has been shown also to catalyze reactions with other sugars. For example, xylitol is a sugar alcohol that is transported through the phloem as a carbon source (Lewis and Smith, 1967; Lemoine et al., 2013). Xylitol can be converted to xylulose and be used in the pentose phosphate pathway to generate glycolytic intermediates as a source of energy. Since the AcDCXR catalyzes the reversible reaction between xylulose and xylitol, the enzyme may provide the aphid an additional mode of generating energy.

Diacetyl/L-xylulose reductases also participates in the reductive metabolism of carbonyls. In this role, the enzyme is considered as a defense mechanism against harmful carbonyls (Nakagawa et al., 2002; Ebert et al., 2015; Yang et al., 2017). These molecules lead to formation of AGEs by reacting with lysine, cysteine and arginine, thus inactivating proteins (Thornalley, 2006; Ahmed and Thornalley, 2007). One of these harmful carbonyls is methylglyoxal which is reactive α-β-dicarbonyl ketoaldehyde. Interestingly, methylglyoxal has been shown to accumulate in a number of plant species under various abiotic stresses (Yadav et al., 2005a; Hossain et al., 2009; Mustafiz et al., 2014; Rahman et al., 2015; Borysiuk et al., 2018). Recently, methylglyoxal has also been implicated in biotic stresses. Increases in methylglyoxal levels were detected in tobacco plants exposed to the bacterium *Pseudomonas syringae*, or the *Mungbean yellow mosaic virus*, or to the fungus *Alternaria alternata* (Melvin et al., 2017). In addition, exogenous application of methylglyoxal in wheat and rice plants upregulated antioxidant and defense-related genes indicating a role for methylglyoxal in plant defense (Kaur et al., 2015; Li et al., 2017). In this work we showed that aphid feeding also enhanced accumulation of methylglyoxal in cowpea and pea, suggesting methylglyoxal also functions in aphid defense. Since methylglyoxal levels in aphid infested leaves were mostly transient, this suggests that aphids are able to counteract methylglyoxal accumulation possibly through AcDCXR activity.

Transient expression of AcDCXR indicates that this enzyme is localized in the plant cell cytoplasm. Likewise, both AcDCXR substrates tested in this study, methylglyoxal and xylitol/xylulose, are also located in the cell cytoplasm. In plants, the pentose phosphate pathway where xylitol/xylulose are used, takes place in both the cytoplasm and plastids. Methylglyoxal is generated in multiple pathways in the cytoplasm and in various organelles (Phillips and Thornalley, 1993; Dennis and Blakeley, 2000; Kruger and von Schaewen, 2003).

The transient expression of AcDCXR increased the fecundity of the cowpea aphid most likely due to its effect on one or both of these two substrates; either by increasing the obtained nutrient content and/or through diminishing defense responses. This increase in fecundity was seen despite no differences in the survival of both adult and nymphal stages of the aphid. Transient or stable overexpression of a number of aphid effectors in various plant species including, Arabidopsis, tomato, pea and *N. benthamiana* also yielded increases in aphid fecundity but no effect on aphid survival suggesting that overexpression of multiple effectors may be needed to observe a pronounced change in aphid survival.

In this work, using a classical and a novel bioinformatics programs, SignalP and EffectorP, respectively, we identified a novel aphid effector, AcDCXR. The functional annotation of DCXR and *in vitro* biochemical analysis of AcDCXR lead us to identify methylglyoxal as a potential novel metabolite involved in aphid defense. Therefore, identification of novel effectors may lead to the discovery of yet unknown defense pathways that may lead to novel approaches to engineer pest/pathogen resistance in crops.

## Data Availability Statement

The datasets generated for this study can be found in MassIVE, https://massive.ucsd.edu/ProteoSAFe/static/massive.jsp, ID: PXD017323

## Author Contributions

IK conceived the project. IK, SD, and AS designed the experiments. JM conducted the experiments. IK, JM, SD, and QC analyzed the data. IK and JM wrote the manuscript with help from all other authors.

## Conflict of Interest

The authors declare that the research was conducted in the absence of any commercial or financial relationships that could be construed as a potential conflict of interest.

## Footnotes

1 https://agops.ucr.edu/soil-mixing
