# Supplementary material for: AcDCXR Is a Cowpea Aphid Effector With Putative Roles in Altering Host Immunity and Physiology
Source: Front Plant Sci. 2020 May 15;11:605. doi: 10.3389/fpls.2020.00605 (PMC7243947; doi:10.3389/fpls.2020.00605)
Supplement: Supplementary file 2 [file Data_Sheet_1.DOCX]

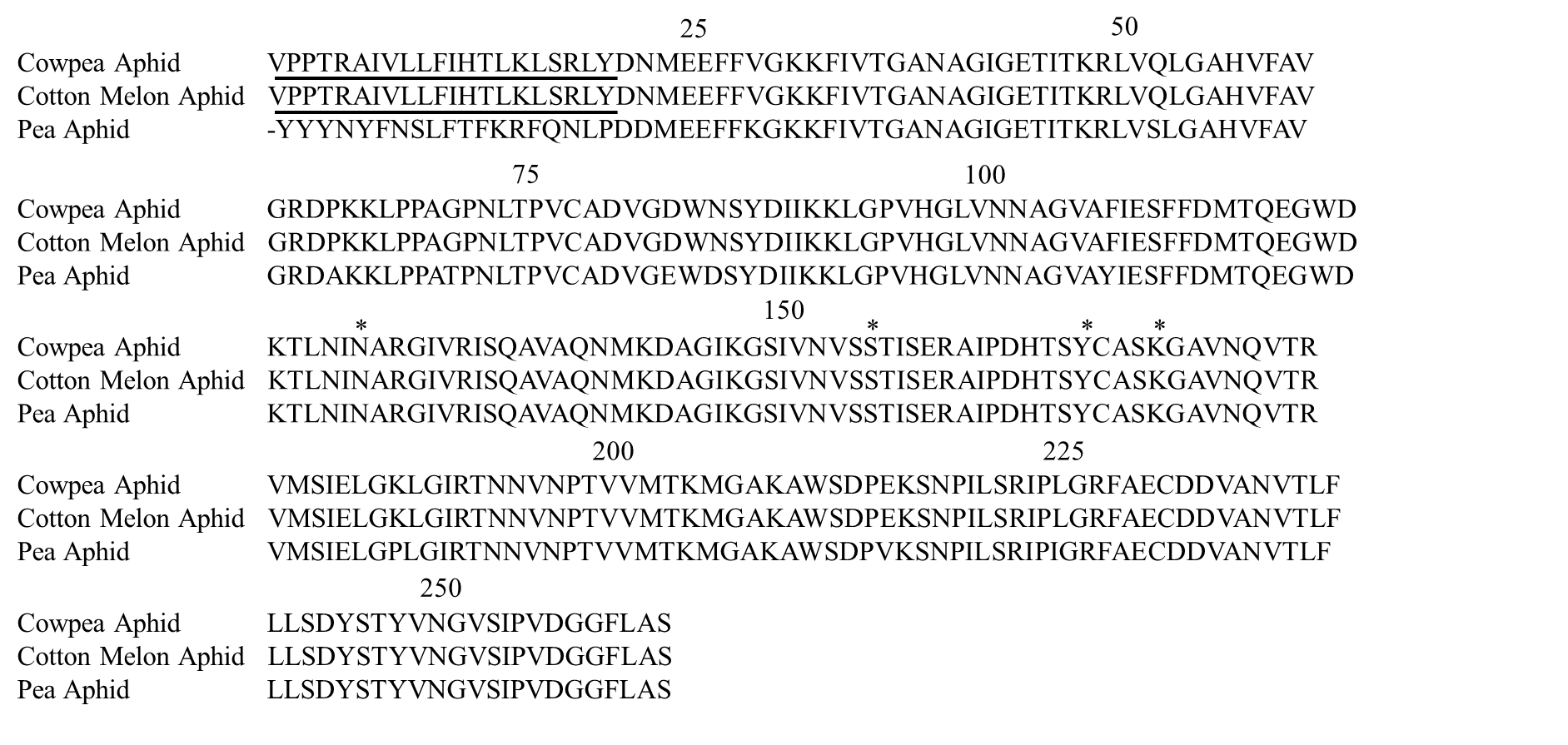


**Supplemental Figure 1. DCXR amino acid sequence alignment.** Alignment of AcDCXR from cowpea aphid (*Aphis craccivora*), and orthologs from the cotton melon aphid (*Aphis gossypii*; XP_027848224.1) and the pea aphid (*Acyrthosiphon pisum*; NP_001119641.1) using Clustal Omega (Sievers et al., 2011). The secretion signal found in the cowpea aphid and cotton melon aphid using SignalP is underlined (Bendsten et al., 2004b; Armenteros et al., 2019). The active site for short-chain dehydrogenases/reductases is made up of a YXXXK motif with an upstream N and S (Marchler-Bauer et al., 2017). The residues forming the active site are indicated with the asterisks.

**REFERENCES**

Armenteros, J.J.A., Tsirigos, K.D., Sonderby, C.K., Peterson, T.N., Winther, O., Brunak, S., et al. (2019). SignalP 5.0 improves signal peptide predictions using deep neural networks. *Nature Biotechnology* 37**,** 420-423. doi: 10.1038/s41587-019-0036-z

Bendtsen, J.D., Nielsen, H., von Heijne, G., Brunak, S. (2004b). Improved prediction of signal peptides: SignalP 3.0. *Journal of Molecular Biology* 340(4) 783-795. doi: 10.1016/j.jmb.2004.05.028.

Marchler-Bauer, A., Bo Y., Han, L., He, J., Lanczycki, C.J., Lu, S., et al. (2017). CDD/SPARCLE: functional classification of proteins via subfamily domain architectures*.* *Nucleic Acids Res.* 45(D1), 200-203. doi: 10.1093/nar/gkw1129.

Sievers, F., Wilm, A., Dineen, D., Gibson, T.J., Karplus, K., Li, W., et al., (2011) Fast, scalable generation of high-quality protein multiple sequence alignments using Clustal Omega. Mol Syst Biol 7 539. doi: 10.1038/msb.2011.75.


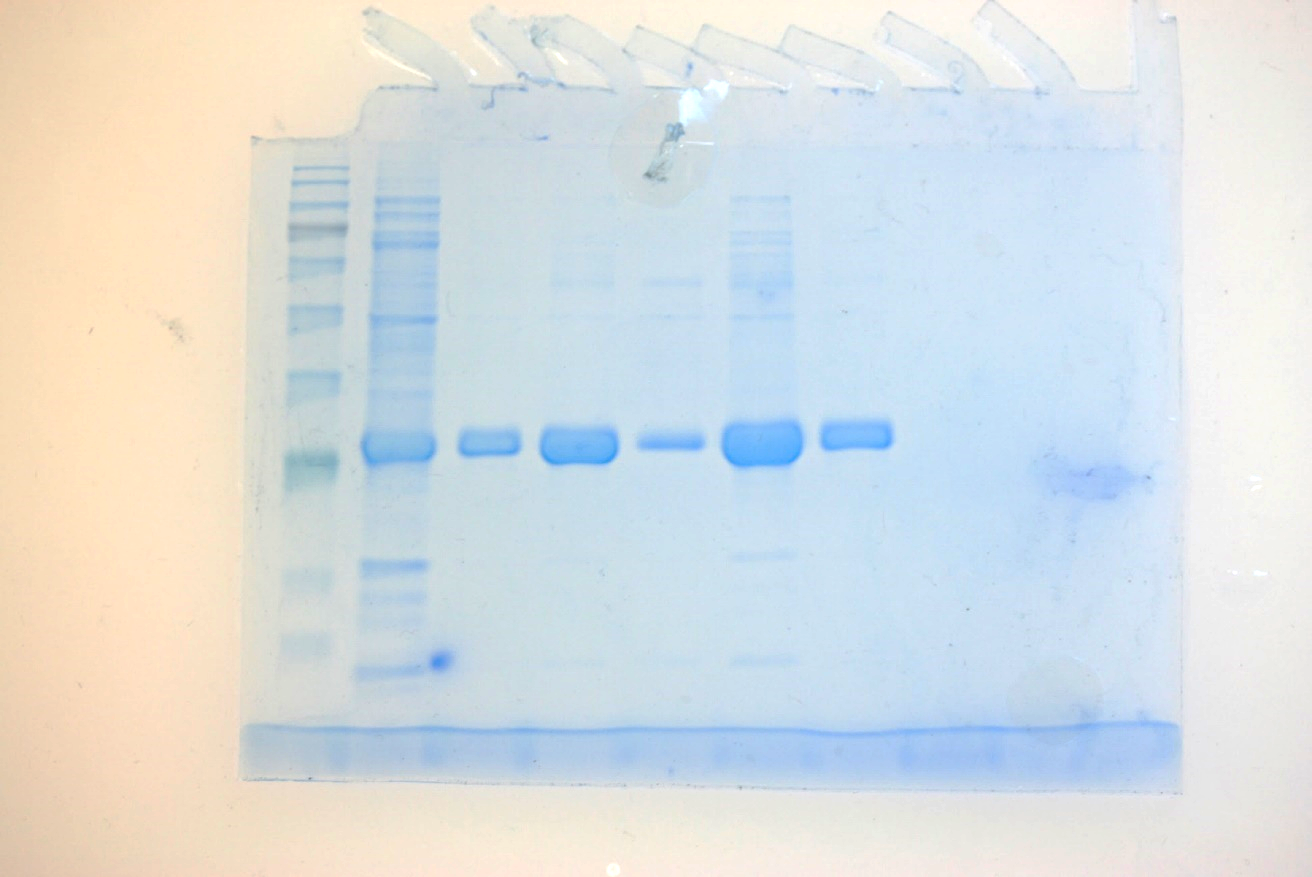


E2

E3

25 kDa

35 kDa

E1

M

**Supplemental Figure 2. Recombinant AcDCXR purified from *E. coli***. pDEST17-AcDCXR was expressed in *E. coli* and induced with 0.5 mM IPTG. Aliquots of the purification products were run on 12% SDS−PAGE. M is the protein standard, E1 and E2 are the first (150 mM imidazole) and second (200 mM imidazole) elutions from the Ni-NTA column. The expected 6xHis tagged AcDCXR size is 28.3 kDa.

**Supplemental Figure 3. Western blot analysis of GFP expression in *Pisum sativum* cv ZP1130.** Leaves were infiltrated with with *Agrobacterium tumenfaciens* containing pEAQ-DEST1-GFP and samples were collected at 2, 3, 5,7, 8, 9, 10 days post infiltration (dpi). Samples were extracted with urea-thiourea lysis buffer and 25 ug of protein was loaded onto 12% SDS-PAGE. GFP was detected with anti-GFP at 1:2000 dilution. Lower panel is Ponceau S Red staining of Rubisco to show protein loading.


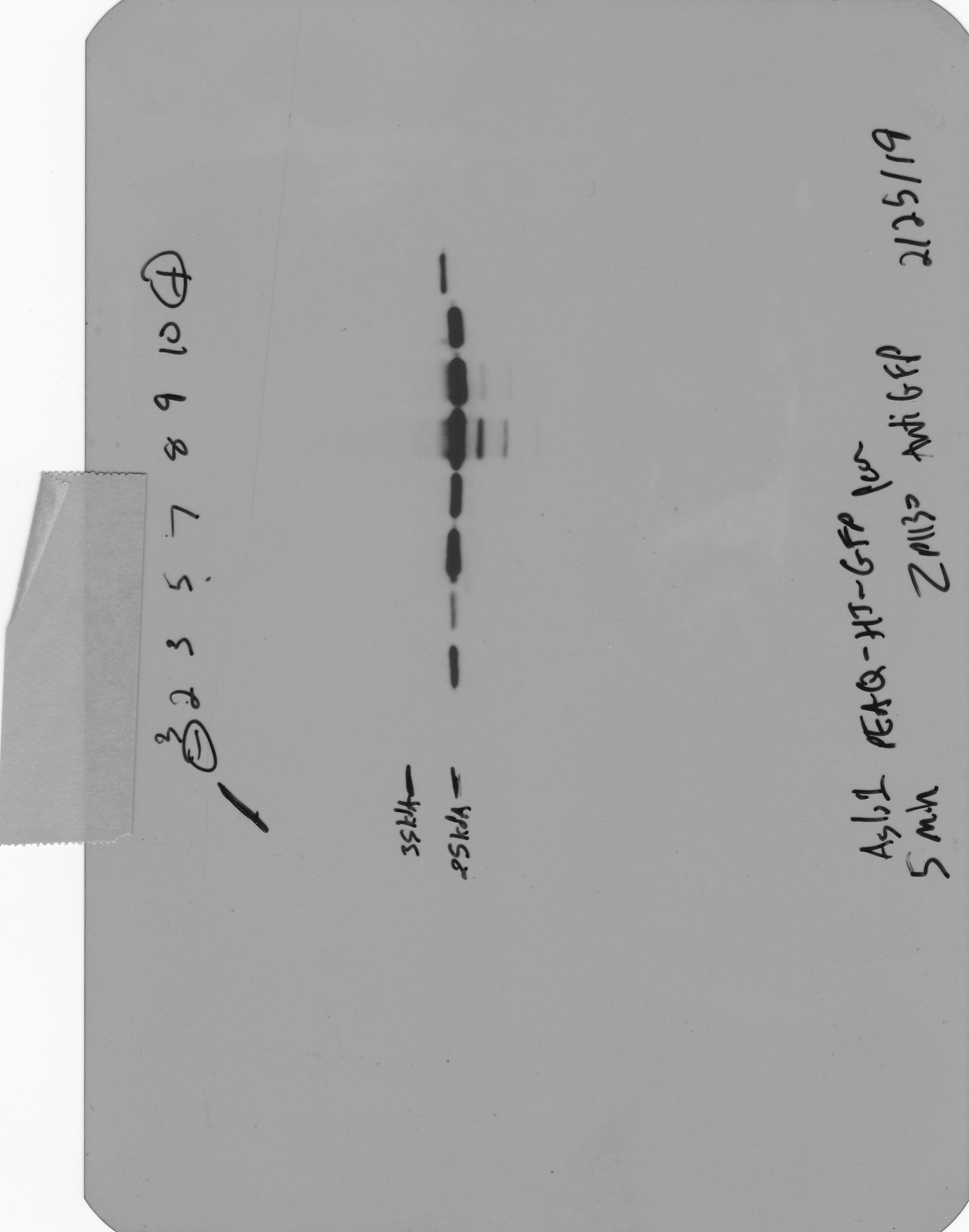


2

3

5

7

8

9

10


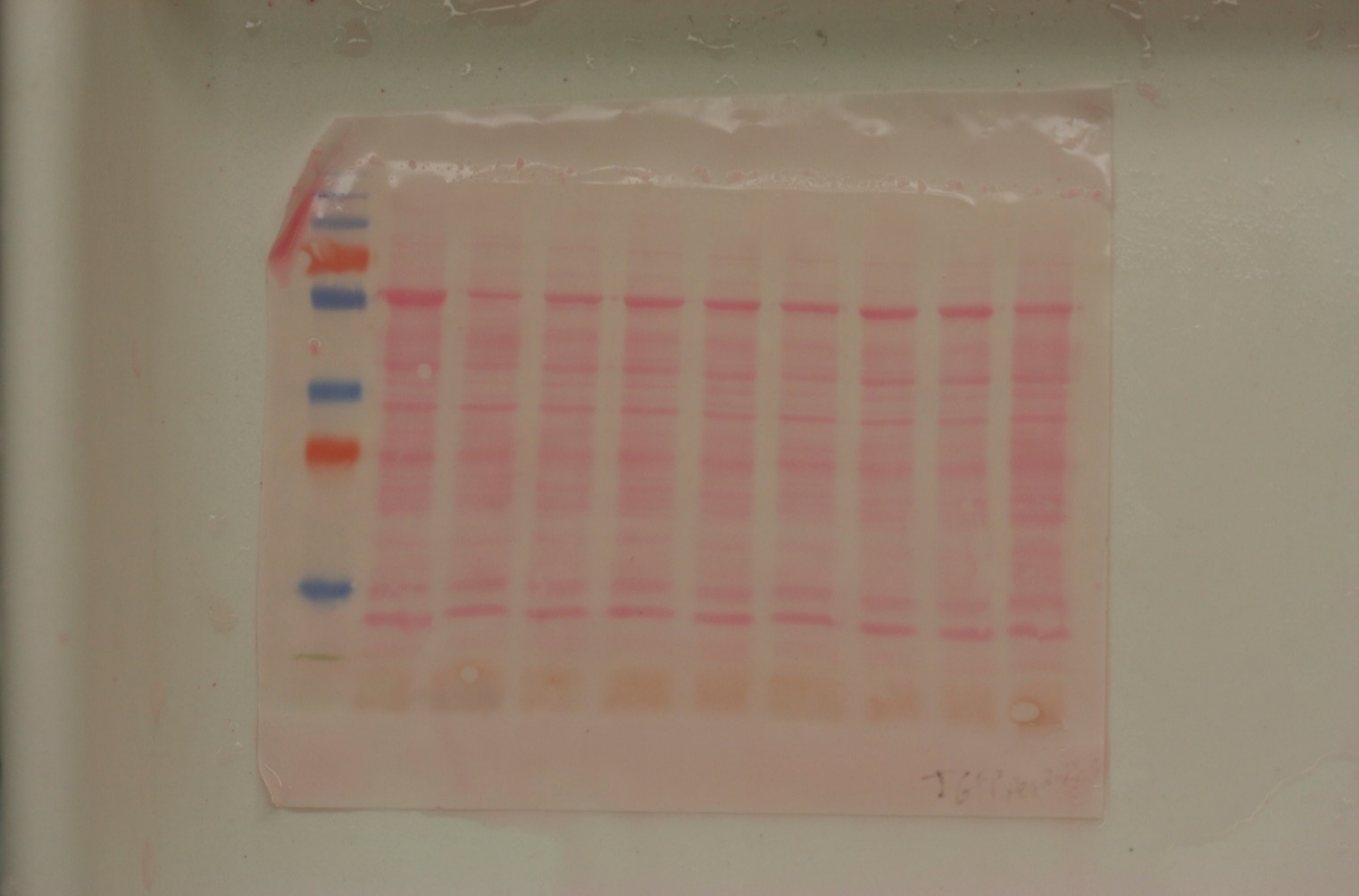


Rubisco

dpi

GFP
